# CPW-Fed Flexible Ultra-Wideband Antenna for IoT Applications

**DOI:** 10.3390/mi12040453

**Published:** 2021-04-17

**Authors:** Sharadindu Gopal Kirtania, Bachir Adham Younes, Abdul Rakib Hossain, Tutku Karacolak, Praveen Kumar Sekhar

**Affiliations:** School of Engineering and Computer Science, Washington State University Vancouver, Vancouver, WA 98686, USA; sharadindu.kirtania@wsu.edu (S.G.K.); bachir.younes@wsu.edu (B.A.Y.); abdul.hossain@wsu.edu (A.R.H.); tutku.karacolak@wsu.edu (T.K.)

**Keywords:** UWB antenna, IoT, inkjet printer, PET, bending analysis, circular-shaped antenna

## Abstract

In this article, an inkjet-printed circular-shaped monopole ultra-wideband (UWB) antenna with an inside-cut feed structure was implemented on a flexible polyethylene terephthalate (PET) substrate. The coplanar waveguide (CPW)-fed antenna was designed using ANSYS high-frequency structural simulator (HFSS), which operates at 3.04–10.70 GHz and 15.18–18 GHz (upper $K_{u}$ band) with a return loss < −10 dB and a VSWR < 2. The antenna, with the dimensions of 47 mm × 25 mm × 0.135 mm, exhibited omnidirectional radiation characteristics over the entire impedance bandwidth, with an average peak gain of 3.94 dBi. The simulated antenna structure was in good agreement with the experiment’s measured results under flat and bending conditions, making it conducive for flexible and wearable Internet of things (IoT) applications.

## 1. Introduction

The integration of advanced technologies in the modern communication framework has accelerated the widespread use of interconnected miniaturized devices in the Internet of things (IoT) [1,2] platform. The IoT applications demand connectivity among devices in a wide variety of applications requiring multiple communication devices [3,4,5,6,7]. For improving system performance and reconfigurability, sometimes lumped equivalent circuit models of an antenna are used in modern communication devices, which may require complex simulation and mathematical analysis [8]. Ultra-wideband (UWB) technology-based devices have the potential to accommodate the demanding needs of connectivity considering the additional constraints of reduced IoT circuit elements, as well as the devices’ size, weight, and budget.

The UWB technology has gained significant attention over the last decade due to its wide bandwidth, high-speed data rate characteristics, power efficiency, non-interfering signals, efficient use of spectrum, secure communication system, and simple circuitry for implementation [9]. For UWB applications, a frequency range of 3.1 to 10.6 GHz, with a bandwidth of 7.5 GHz, has been allocated by the Federal Commission of Communication (FCC), USA [10,11,12]. UWB antennas form an integral part of wireless body area networks (WBAN) and IoT devices for their compactness and simplicity [13]. The UWB antennas find niche applications where low-cost wireless sensors are used for continuous data transmission and low-radiated power characteristics, predominantly in wearable or flexible IoT devices [14,15,16,17].

Numerous UWB antenna designs on flexible substrates exist in the literature [1,3,18,19,20,21,22,23]. Mustaqim et al. designed a rectangular UWB antenna on FR4 and a denim textile substrate, operating in the 2.9 to 11 GHz band for wearable IoTs [1]. Saha et al. developed a CPW-fed UWB antenna on a paper substrate, operating in 3.2–30 GHz with considerable gain for IoT applications [3]. Wang et al. presented a flexible UWB antenna fabricated using a surface modification and an in situ self-metallization technique on a polyimide substrate, which covered a 1.35 to 16.40 GHz band [18]. Fang et al. developed a graphene-assembled film (GAF)-based compact and low-profile ultra-wide bandwidth (UWB) antenna covering 4.1–8.0 GHz wearable applications [19]. Natale and Giampaolo presented a reconfigurable UWB antenna for WBAN on fabric materials [20]. Zhang et al. proposed and analyzed a flexible UWB antenna, covering 3.06 to 13.58 GHz, both in flat and bent states [22]. A UWB antenna operating from 3.1 GHz to 11.3 GHz was developed by El Gharbi et al., on a felt textile substrate [23]. Table 1 provides a concise literature review of the different kinds of UWB antennas designed on rigid and flexible substrates in recent years.

With an aim to improve the UWB antenna’s performance characteristics, this article explores an inkjet-printed circular-shaped UWB monopole antenna on PET for IoT applications. The flexible UWB antenna is designed to operate in the 3.04–10.70 GHz and 15.18–18 GHz (upper $K_{u}$ band). A prototype of the proposed UWB antenna was fabricated to measure its reflection coefficient ($S_{11}$), compared with the simulated ones in flat and bent conditions. Other antenna parameters such as gain, radiation pattern, and radiation efficiency were also simulated in the ANSYS and discussed in the article. The manuscript contains the following sections: Section 2 and Section 3 cover the parametric study results and fabrication mechanism for the proposed UWB antenna. Section 4 compares the measured $S_{11}$ with the simulated ones and includes the simulated 2D radiation patterns and other antenna parameters along with the antennas’ bending effects under different radii. Lastly, the conclusions are drawn in Section 5.

## 2. Antenna Design and Analysis

A commercially available PET substrate with a dielectric constant of 3.2, a loss tangent of 0.022, and a thickness of 135 µm was used for designing the proposed antenna.

**Figure 1:**
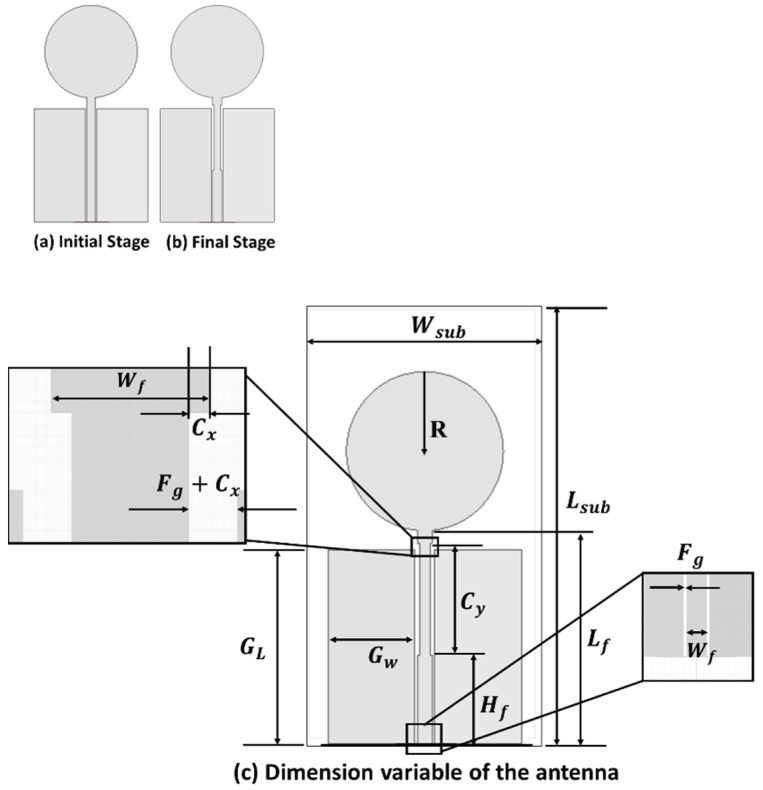
The evolution of the proposed antenna design, with dimension variables (**a**) Initial stage, (**b**) Final stage and (**c**) Dimensions variables of the proposed antenna.

The dimension of the antenna was 47 mm ×25 mm ×0.140 mm. The evolution of the proposed antenna design is shown in Figure 1.

**Figure 2:**
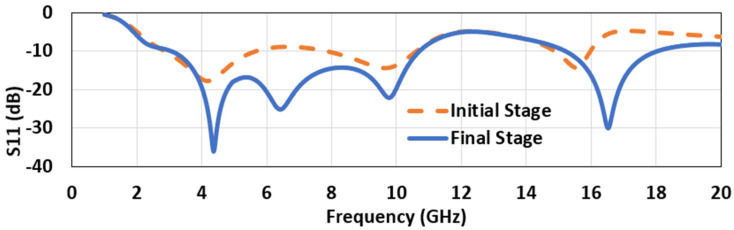
The reflection coefficient plot of the antenna’s design stages.

The design process started with a conventional CPW-fed (50 Ω) circular-shaped monopole antenna, as shown in Figure 1a. The ground and feedline gap and parameters related to the feedline gap ($F_{g}$), feedline width ($W_{f}$), and feedline cut structure ($C_{x},C_{y}$) controlled the $S_{11}$ parameter of the antenna. The feedline was modified in the second and final stage (Figure 1b) and was slightly cut from the side adjacent to the patch. Figure 1c shows the dimension of the antenna with different variables. The ANSYS HFSS (Canonsburg, PA, USA) -simulated final dimensions are given in Table 2. Less than −10 dB bandwidth from 3.04 GHz to 10.70 GHz and 15.18 GHz to 18 GHz was obtained from this design (Figure 2).

**Figure 3:**
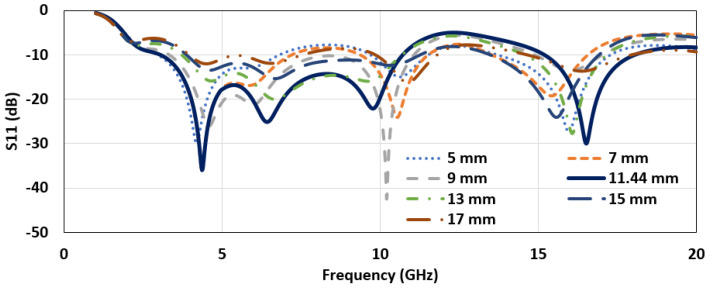
The reflection coefficient plot for different values of cut length of the feedline (depth, *C_x_* = 0.2 mm).

The implementation of the designed antenna is driven by investigating the parameters that impact the frequency response. The parametric study was started by introducing a side cut on the feedline structure and varying the cut length and depth. Figure 3 illustrates the reflection coefficient versus the frequency plot for different values of the cut length from the feedline. A smaller bandwidth was observed for smaller cut lengths. For 11.44 mm, a cut length along the feedline gives the highest bandwidth with acceptable impedance performance.

**Figure 4:**
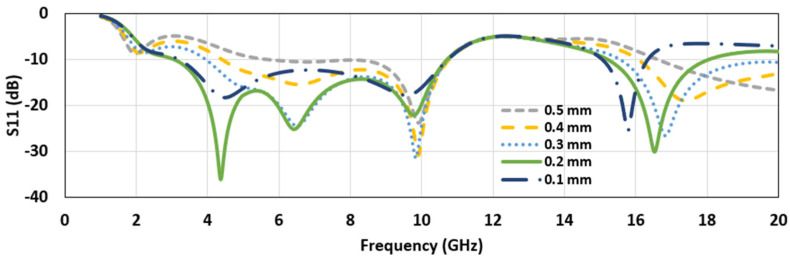
The reflection coefficient plot ($S_{11}$) for different feedline cut depths (*C_x_*) (cut length, *C_y_*= 11.44 mm).

Increasing the cut length further fails to give an acceptable reflection coefficient. Next, keeping the cut length fixed at 11.44 mm, the depth of the cut was changed between 0.1 mm and 0.5 mm. The plot of the reflection coefficient for different depths ($C_{x}$) of the side cut are shown in Figure 4. Increasing the depth of the feedline cut from 0.1 mm to 0.2 mm improved the impedance-matching bandwidth; however, further increases in $C_{x}$ deteriorated the condition. Thus, 0.2 mm was used as the optimized value for the cut depth from two sides of the feedline.

**Figure 5:**
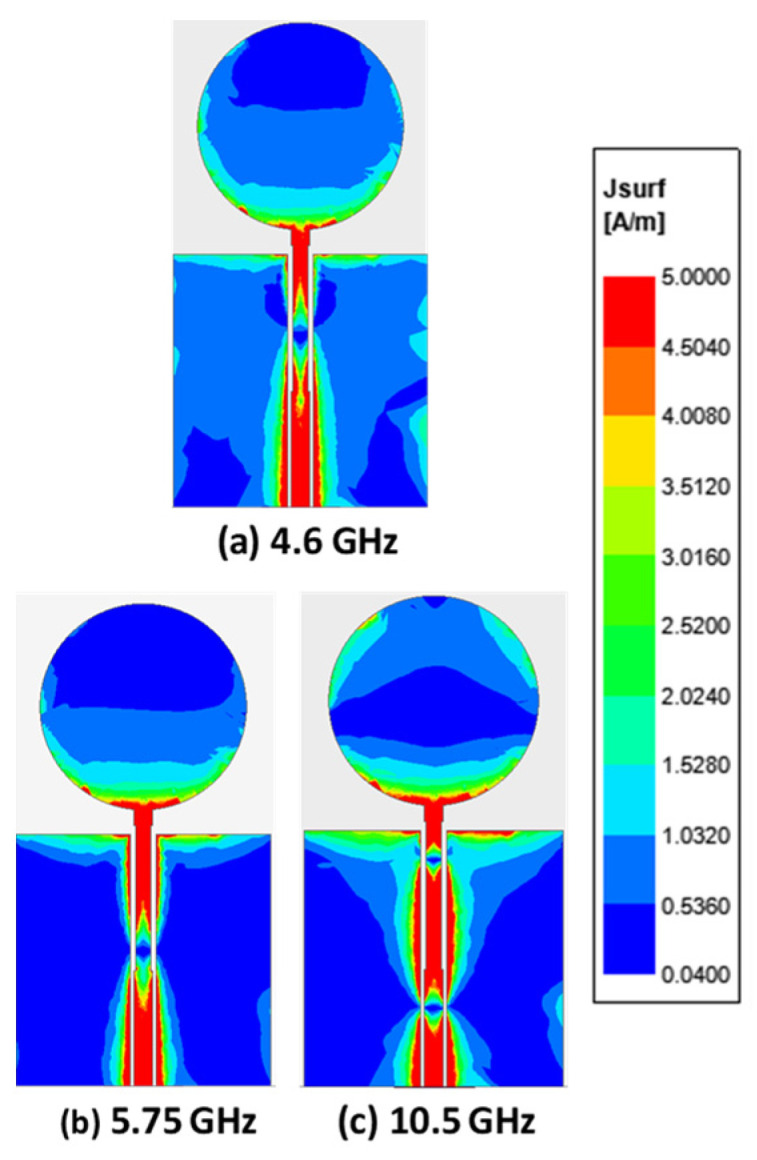
The current distribution plot of the proposed antenna at different resonant frequencies (**a**) 4.6 GHz, (**b**) 5.75 GHz and (**c**) 10.5 GHz.

From the parametric studies, it appeared that the tuning of the side cut length $C_{y}$, depth of the side cut $C_{x}$, and the width of the feedline strip, $W_{f}$, generated a wider bandwidth. Such an observation can be justified from the current distribution plot (Figure 5) of the antenna at the three resonant frequency points of 4.6 GHz, 5.75 GHz, and 10.5 GHz. The maximum current density appeared to be concentrated at the feedline and feeding neck antenna, indicating that the tuning of the parameters $C_{y}$, $C_{x}$, and $W_{f}$ generates a wider impedance bandwidth.

## 3. Fabrication of the Antenna

**Figure 6:**
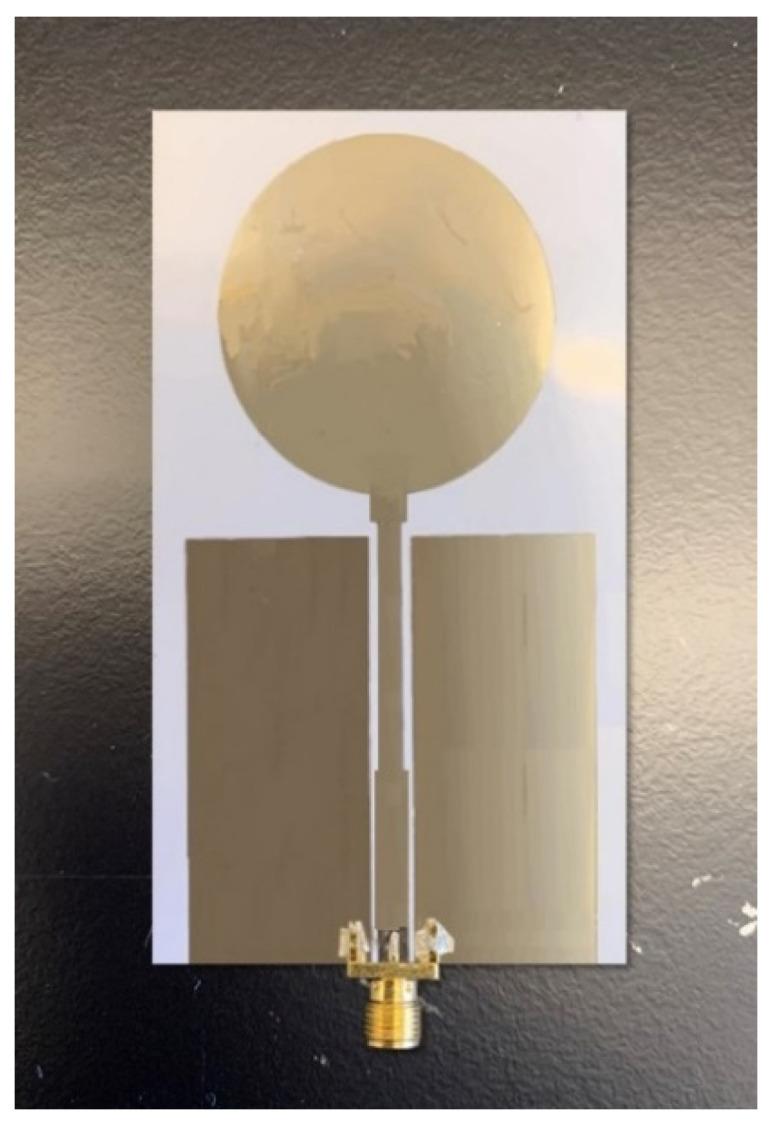
Inkjet-printed UWB antenna.

The designed free-space antenna was printed using a Fujifilm Dimatix 2831 Inkjet printer (DMP, Fujifilm, Santa Clara, CA, USA), which offers an accurate printing, using 1 pL and 10 pL volume cartridges. For the fabrication of the antenna, a 10 pL cartridge was used, which has 16 nozzles of diameter 21 µm, and was driven by the piezoelectric element of the Dimatix printer. A commercially available silver (Ag) nanoparticle ink, JS102A with a 40 wt% Ag content, viscosity of 8 to 12 cp, and surface tension of 19–30 dyne/cm was used for the experiment. The ink was purchased from Novacentrix, Austin, TX, USA. Optimized printing parameters were adopted from an earlier study [39] to print the antenna. The printed antenna samples were sintered on a hotplate at 120° temperature to create a compact silver layer and generate conductivity. An Agilent PNA-LN5230C vector network analyzer (VNA) was used for the validation of the fabricated antenna samples. Figure 6 shows the inkjet-printed antenna on the PET substrate.

## 4. Results and Discussion

**Figure 7:**
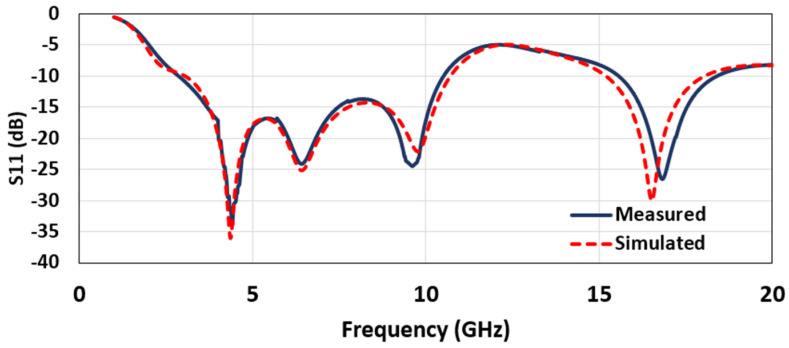
The simulated and measured return loss (dB) of the proposed antenna.

The reflection coefficient ($S_{11}$) from the simulation and the antenna measurement is shown in Figure 7. It is evident that the design had four resonance frequencies: 4.36, 6.42, 9.76, and 16.52 GHz. The operating bandwidths around these frequencies overlapped, leading to a -10 dB bandwidth from 3.04 to 10.70 GHz (FBW 111.66%) and 15.2 to 18 GHz, which covered the partial/upper $K_{u}$ band. This result seems quite interesting, as the same UWB antenna may also be used for the upper $K_{u}$ band application. However, in this research, we mainly emphasize the UWB part. The measurement was in good agreement with the simulation, with a minor shift in resonant frequencies. The frequency shifts are ascribed to the minor imperfections in fabrication, SMA connector losses, the effectiveness of the silver-epoxy, and the measuring environment. However, the achieved bandwidth covers the target UWB frequency band application.

**Figure 8:**
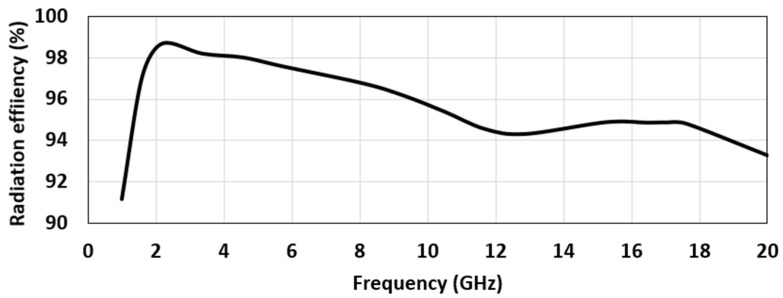
The simulated radiation efficiency plot as a function of frequency.

**Figure 9:**
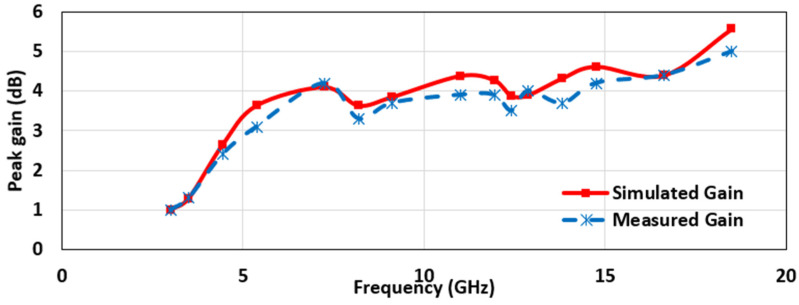
The simulated and measured peak gain plot as a function of frequency.

The peak radiation efficiency in the operating bandwidth was found to be 98%, at 4.56 GHz, and the average radiation efficiency was 95.7%, over the entire bandwidth (Figure 8). The radiation efficiency appears to decline with an increase in the frequency. The reason for such an observation is that the patch’s geometry becomes larger at the corresponding wavelength, which is consistent with the earlier literature [3,40]. Figure 9 indicates the simulated peak gain variations as a function of frequency. As observed, the antenna had an average peak gain of 3.89 dB. Noticeably, the gain plot indicated an increasing trend with an increasing frequency. The highest peak gain for the UWB region was seen at the highest frequency of 10.6 GHz, which was 4.25 dB, and for the $K_{u}$ band, which was 5.7 dB, at 18 GHz. The gain increased with the increasing frequency, due to the antenna’s large electrical size at that frequency, compared with its size at other lower operating frequencies (Figure 9). The gain of the antenna was also measured by placing two identical antennas, kept in the same line of sight and separated with a far-field distance, $\frac{2D^{2}}{\lambda }$, where D is the maximum dimension of the antenna, and λ is the wavelength of the highest frequency. Then, the well-known Friis power transmission equation was used to calculate the gain in the far-field region:

(1) $$\frac{P_{r}}{P_{t}}={\left|S_{21}\right|}^{2}=G_{t}G_{r}{\left(\frac{\lambda }{4\pi R}\right)}^{2}$$

where the ratio of the received power to input power, $\frac{P_{r}}{P_{t}}$, equals the transmission coefficient ${\left|S_{21}\right|}^{2}$, which was taken from the network analyzer. Here, as we used two identical antennas; hence, $G_{t}\;\mathrm{and}\;G_{r}$ are equal. The distance, R, was kept greater than the far-field distance. The measured gain had a good agreement with the simulated response. The scatterings and reflections from the surrounding environment caused the difference between the simulated and the measured gains (Figure 9).

**Figure 10:**
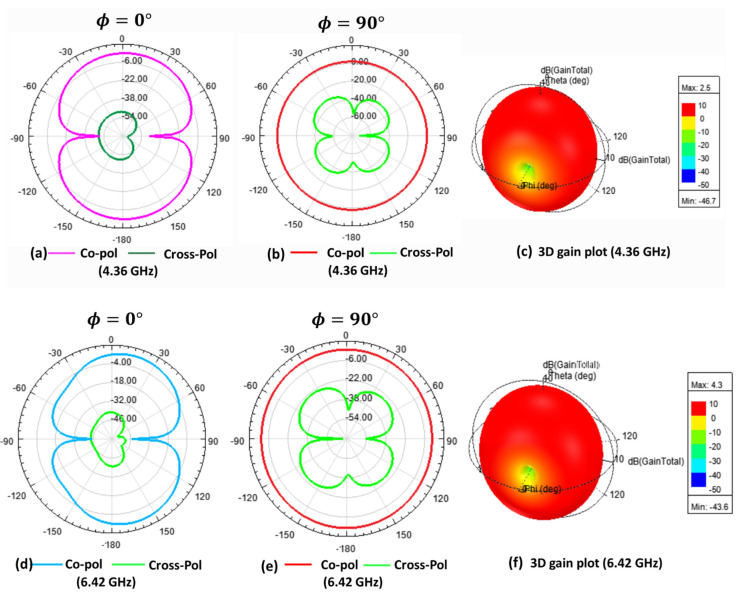
The simulated co- and cross-polarization and 3D gain plot: (**a**) The x-z plane; (**b**) The y-z plane; (**c**) The 3D gain plot, at 4.36 GHz; (**d**) The x-z plane; (**e**) The y-z plane; and (**f**) The 3D gain plot, at 6.42 GHz.

**Figure 11:**
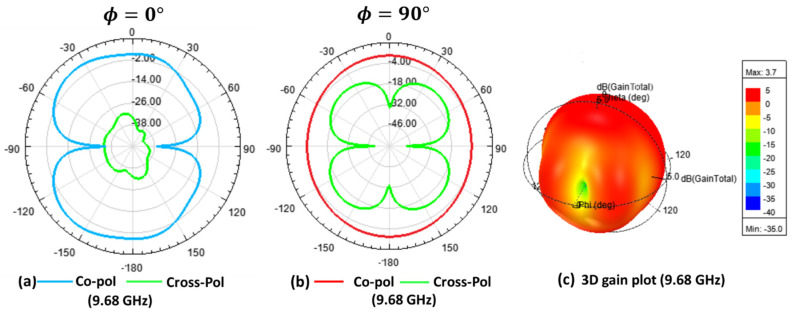

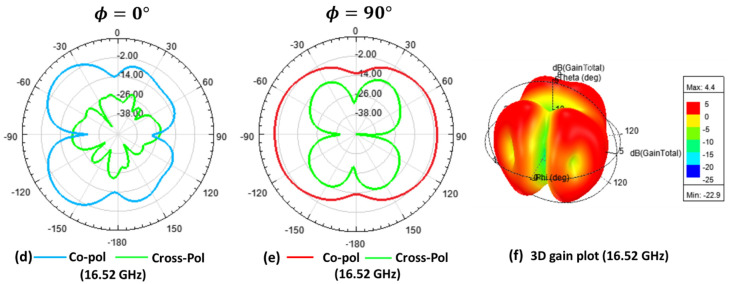
The simulated co- and cross-polarization and 3D gain plot: (**a**) The x-z plane; (**b**) The y-z plane; (**c**) The 3D gain plot at 9.68 GHz; (**d**) The x-z plane; (**e**) The y-z plane; and (**f**) The 3D gain plot at 16.52 GHz.

In addition to investigating the return loss and radiation efficiency, the antenna radiation pattern was also examined. The proposed antenna’s simulated radiation pattern (Figure 10 and Figure 11) matches with the earlier reported work [41], which confirms the design’s validity. Generally, IoT applications require an omnidirectional radiation pattern [42]. From the simulation of co-polarized, cross-polarized field plots and the 3D gain plot, it was observed that the antenna presents omnidirectional characteristics. Besides, the x-z plane’s cross-polarization value was 12 to 45 dB lower than the co-polarized values, whereas the cross-polarization on the y-z plane was 5 to 30 dB lower than the co-polarized values for the whole application band. Due to the lack of instrumentation, the experimental radiation pattern could not be measured. Figure 10 shows the radiation pattern for 4.36 and 6.42 GHz. Figure 11 shows the radiation pattern for 9.68 and 16.52 GHz.

**Figure 12:**
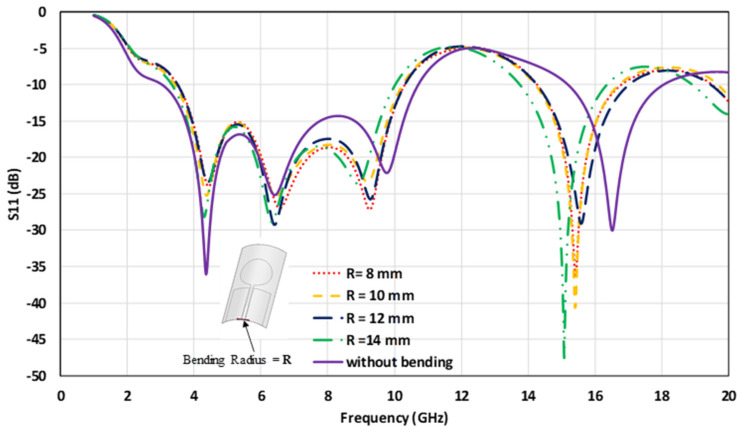
The simulated bending impact on the antenna’s return loss.

The antenna’s performance attributes mainly to the resonant frequency and the return loss, which need to be investigated under bent conditions in order to assess real-time IoT application suitability. Figure 12 shows the effect of bending at different bending radii, R, on the antenna’s simulated return loss. Based on the simulation, a slight shift in frequencies was observed in the operating region for the bent antenna, compared with the flat configuration. Such an observation is attributed to the fact that bending increases the antenna’s resonant length, which results in shifting the resonance toward lower frequencies. From Figure 12, it is worth mentioning that, with the increase in the bending radius, the resonant frequency in the 15–17 GHz changed non-uniformly. This can be due to the multiple effects of bending on the $S_{11}$ parameter. In [43], the authors showed a non-linear relationship between the frequency and the bending radius, both analytically and with experimental validation. As mentioned, bending can change the transmission line’s effective dimension; it can also change the gap between the feedline and the ground, which, in turn, changes the antenna’s impedance matching [44]. As the bending deformed the antenna’s ground (both sides) slightly, it could also cause the resonant frequency shift, since the vicinity of the ground affected the current density of the antenna, which controlled the $S_{11}$ parameter [45]. Therefore, multiple phenomena can happen during bending, which make it difficult to predict the actual behavior trend of the antenna’s $S_{11}$ parameter. Thus, non-linear shifts arise, even though the bending radius changes linearly. The antenna’s bending characteristics were investigated by placing it around a cylindrical object of 8 cm radius with permittivity, $\varepsilon_{r}$ = 2.7.

**Figure 13:**
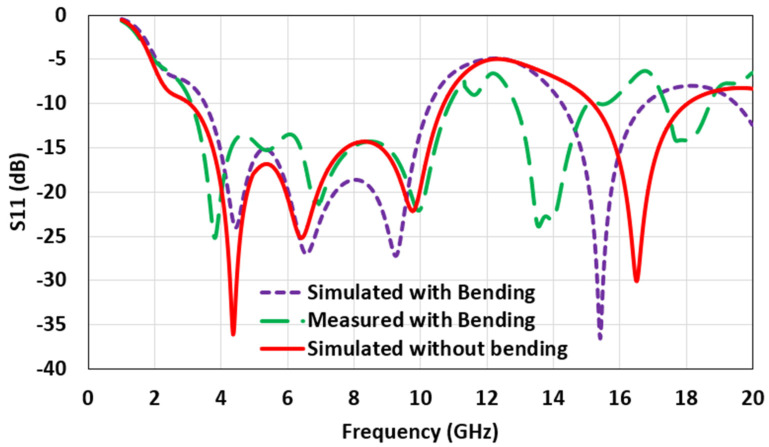
The measured bending impact on the antenna’s return loss for the bending radius of 8 mm.

Figure 13 shows the comparison of the simulated and the measured bending impact on the return loss for the bending radius of 8 mm. A frequency shift was observed during the testing, indicating a degradation in impedance matching in higher operating frequencies. This is attributed to the higher permittivity of the used cylindrical structure, compared with the simulation ($\varepsilon_{r}$ = 1). An increase of $\varepsilon_{r}$ decreases the resistive part of input impedance, and the reactance part becomes capacitive [46,47]. Nonetheless, the antenna maintains the desired application bandwidth for the UWB region, ensuring ample flexibility and mechanical sturdiness, and meeting practical IoT application requirements.

## 5. Conclusions

In this article, a flexible UWB antenna on a PET substrate was designed and implemented. The designed antenna was derived from a CPW feedline by loading a circular radiator and cutting a slot on the feedline, in order to enhance the bandwidth. The antenna was designed not only for the flexible application of IoT devices, but also for the free space at ultrawide frequency bands and partial X-bands. The designed antenna exhibited an improved radiation efficiency and FWB, compared with the other flexible UWB antennas reported in the literature. The free space UWB antenna was fabricated and validated through simulation and measurement results. The antenna had an omnidirectional radiation pattern, with a peak gain of 4.25 dB at 10.6 GHz, in the ultra-wideband, and a peak gain of 5.7 dB at 18.5 GHz, in the partial $K_{u}$ band. Due to limited infrastructure, we were not able to perform the radiation pattern measurement. Nonetheless, the antenna’s bending test indicated an electrical and mechanical integrity maintenance, making it an attractive candidate for IoT devices.

## Figures and Tables

**Table 1:** Literature summary of the performance of different CPW-fed antennas for wide frequency bands.

Reference	$\varepsilon_{r}$	Flexibility	Size (mm^2^)	Operating Range (GHz)	FBW %	Peak Gain (dB)	Material
Rabiul et al. [24]	3.2	✓	34 × 25	1.66–16.1	188.5	4.91	PET Paper
Lakrit et al. [25]	2.08	✓	45 × 35	1.20–13.0	166.2	5.40	Teflon
Li et al. [26]	2.65	✓	32 × 24	2.70–12.0	-	3.80	-
Chen et al. [27]	3.00	✓	23 × 20.2	3.00–20.0	-	4.40	Tape
Khaleel et al. [28]	3.40	✓	47 × 33	2.20–14.3	-	4.95	Kapton polyimide
Zhang et al. [22]	3.50	✓	38 × 30.4	2.85–19.4	-	4.15	Kapton polyimide
Varkiani and Afsahi [29]	1.65	✓	23.5 × 22	3.20–16.3	135.0	-	Cotton layer
Wang and Arslan [30]	3.20	✓	26 × 35.5	3.10–10.6	-	-	Polyester
Liu and Kao [31]	4.40	✕	24 × 31	3.05–12.9	-	4.70	FR4
Das et al. [32]	3.20	✕	75 × 63	3.10–10.6	111.4	3.50	Rogers RO4232
Elmobarak et al. [33]	2.70	✓	40 × 40.5	3.30–12.0	-	4.90	Composite laminate
Jalil et al. [34]	1.35	✓	30 × 40	3.00–12.0	-	-	Fleece
Hakimi et al. [35]	3.24	✓	45 × 30	3.15–30.0	164	4.80	PET
Nie et al. [36]	3.66	✕	110 × 120	3.00–10.00	-	6.00	Rogers RO4350B
Shih-Hsun Hsu and Kai Cheng. [37]	2.90	✓	70 × 70	3.00–11.0	-	5.50	Rogers 3850
Kumar and Gupta [38]	3.48	✓	25 × 45	3.00–11.0	-	4.75	RO4350b

**Table 2:** The proposed free-space antenna dimensions.

Parameters	Dimensions (mm)
$L_{sub}$	47
$W_{sub}$	25
$G_{L}$	19.84
$G_{W}$	8.94
$L_{f}$	21.79
$W_{f}$	1.52
R	8.1
$C_{y}$	11.44
$C_{x}$	0.2
$H_{f}$	9.06
$F_{g}$	0.27
